# Dental Notes

**Published:** 1899-09

**Authors:** H. H. Shapard


### Dental Notes.

H. H. SHAPARD, D. D. S.

---

One of the best counter-irritants for peri-cementitis is turpentine.

---

You cannot have acute peri-cementitis with a living pulp, but you can have chronic peri-cementitis without an alveolar abscess.

---

About the shortest and simplest definition for an alveolar abscess is Dr. Darby's, which is, that it is the superative stage of peri-cementitis.

---

People who mind everybody's business generally have difficulty in making their own business mind them; so don't speak about your fellow practitioner unless you can speak words of praise.

---

At the last meeting of the State Dental Association the subject of antiseptic mouth washes was discussed, and many of the dentists present complained that on account of most of the washes on the market, such as borolyptol, Wampole's solution, etc., being so expensive that they experienced a great deal of trouble in geting the poorer classes of their patients to use them. This trouble can be easily obviated by using Dr. Trueman's prescription, which I have been using since I began the practice of dentistry, and consider one of the best as well as one of the cheapest mouth washes in use. You can make it at a cost of a little less than three cents per ounce. I generally make a gallon at a time and keep it on hand, and when necessary give it to my patients, or add a few cents to their bills to cover cost of same. Below I give the formula, and I would advise those not having tried it to do so:

℞ Hydronaphthol....................gr. xv

Alcohol....................℥j

Aqua dist'd....................℥j

M. Sig.: Teaspoonful in glass of water as a mouth wash.

PAINLESS PULP EXTIRPATION.

℞ Formolin....................1 part
Absolute alcohol....................5 parts

M. Sig.: Pulverize a few crystals of cocaine, saturate the smallest pellet of spunk or cotton with the above solution and gather upon it a little of the powdered cocaine, apply directly to the pulp and after carefully filling the cavity with an unvulcanized rubber, produce a gentle and continuous pressure with a ball burnisher, increasing the force as absence of pain indicates progress of the obtruding influence. Keep this up for several minutes, when the pulp chamber can be thoroughly opened, and the pulp removed generally absolutely without pain. The smallest quantity that can be handled is sufficient.—*O. L. Hertig, "Items of Interest."*

